# Cost analysis of antibiotic therapy versus appendectomy for treatment of uncomplicated acute appendicitis: 5-year results of the APPAC randomized clinical trial

**DOI:** 10.1371/journal.pone.0220202

**Published:** 2019-07-25

**Authors:** Jussi Haijanen, Suvi Sippola, Risto Tuominen, Juha Grönroos, Hannu Paajanen, Tero Rautio, Pia Nordström, Markku Aarnio, Tuomo Rantanen, Saija Hurme, Paulina Salminen

**Affiliations:** 1 Division of Digestive Surgery and Urology, Turku University Hospital, Turku, Finland; 2 Department of Surgery, University of Turku, Turku, Finland; 3 Department of Public Health, University of Turku, Turku, Finland; 4 University of Namibia, Windhoek, Namibia; 5 Department of Surgery, Mikkeli Central Hospital, Mikkeli, Finland; 6 Department of Surgery, Oulu University Hospital, Oulu, Finland; 7 Division of Surgery, Gastroenterology and Oncology, Tampere University Hospital, Tampere, Finland; 8 Department of Surgery, Jyväskylä Central Hospital, Jyväskylä, Finland; 9 Department of Surgery, Kuopio University Hospital, Kuopio, Finland; 10 Department of Biostatistics, University of Turku, Turku, Finland; Vita Salute University of Milan, ITALY

## Abstract

**Background:**

The efficacy and safety of antibiotic treatment for uncomplicated acute appendicitis has been established at long-term follow-up with the majority of recurrences shown to occur within the first year. Overall costs of antibiotics are significantly lower compared with appendectomy at short-term follow-up, but long-term durability of these cost savings is unclear. The study objective was to compare the long-term overall costs of antibiotic therapy versus appendectomy in the treatment of uncomplicated acute appendicitis in the APPAC (APPendicitis ACuta) trial at 5 years.

**Methods and findings:**

This multicentre, non-inferiority randomized clinical trial randomly assigned 530 adult patients with CT-confirmed uncomplicated acute appendicitis to appendectomy or antibiotic treatment at six Finnish hospitals. All major costs during the 5-year follow-up were recorded, whether generated by the initial visit and subsequent treatment or possible recurrent appendicitis. Between November 2009 and June 2012, 273 patients were randomized to appendectomy and 257 to antibiotics. The overall costs of appendectomy were 1.4 times higher (p<0.001) (€5716; 95% CI: €5510 to €5925) compared with antibiotic therapy (€4171; 95% CI: €3879 to €4463) resulting in cost savings of €1545 per patient (95% CI: €1193 to €1899; p<0.001) in the antibiotic group. At 5 years, the majority (61%, n = 156) of antibiotic group patients did not undergo appendectomy.

**Conclusions:**

At 5-year follow-up antibiotic treatment resulted in significantly lower overall costs compared with appendectomy. As the majority of appendicitis recurrences occur within the first year after the initial antibiotic treatment, these results suggest that treating uncomplicated acute appendicitis with antibiotics instead of appendectomy results in lower overall costs even at longer-term follow-up.

## Introduction

Out of the more than 200 million annual surgical procedures performed globally, appendectomy is one of the most common incurring significant health care costs.[1–3] Appendectomy has been the standard treatment for all appendicitis cases for over a century, even though both current epidemiological and clinical data suggest that there may in fact be two different forms of acute appendicitis. These two forms with different disease severity, i.e. uncomplicated and complicated acute appendicitis, appear to be distinct entities instead of consecutive events.[4] Complicated acute appendicitis, defined often as a finding of perforation, appendicolith, abscess, or a suspicion of a tumor[5–7] still requires urgent surgical treatment with the exception of cases presenting with a periappendicular abscess, which are often initially managed conservatively. However, the clinical course of most (70–80%) acute appendicitis cases is uncomplicated. Increasing short-term evidence from randomized trials[6, 8–10] and prospective cohort studies[11, 12] shows that antibiotic therapy for uncomplicated acute appendicitis is a safe and viable treatment alternative. These short-term results have recently been confirmed at 5-year follow-up of the randomized APPAC (APPendicitis ACuta) trial comparing appendectomy with antibiotic therapy in the treatment of CT-confirmed uncomplicated acute appendicitis.[13] Uncomplicated acute appendicitis may also resolve with only symptomatic treatment[14] similar to uncomplicated acute diverticulitis.[15] These findings could further call into question the need for emergency appendectomy for all uncomplicated acute appendicitis patients.[3]

The fundamental differences between antibiotic therapy and surgery as the primary treatment options for uncomplicated acute appendicitis result in the challenge of using comparable definitions of treatment success. The assessment of the optimal treatment paradigm for uncomplicated acute appendicitis cannot be solely based on outcome definition of treatment success defined by risk of recurrent appendicitis as antibiotics will never reach the definitive treatment efficacy of appendectomy in that respect. Future appendectomy may not be a valid primary outcome measure either since about 30% of patients treated with antibiotics will get recurrence and potential protocol stated surgery as with the standard appendectomy approach, 100% of patients will get surgery. Therefore, the comprehensive assessment of the best possible treatment option should also include outcomes independent of the compared treatment strategies, i.e. treatment related morbidity, time to recovery, post-intervention pain, along with patient related factors including patient preference at her or his current situation, and also treatment costs. The economic evaluation of the APPAC trial 1-year follow-up showed 1.6 times higher overall costs for appendectomy taking into account all costs whether generated by the initial visit and subsequent treatment or possible recurrent appendicitis.[16]

Appendicitis recurrence after initial successful antibiotic therapy at long-term follow-up is an important question, as the costs accrued from surgical treatment of the recurrences will reduce the original cost advantage of antibiotics versus surgery. Using a decision tree model in the US context, Wu et al. estimated in their report that a recurrence rate as high as 56% for antibiotic treated patients would be the cut-off point after which initial operative treatment becomes the most cost effective treatment strategy for uncomplicated acute appendicitis.[17] In the APPAC trial 5-year follow-up[13], the majority of the antibiotic group patients (70/100) undergoing subsequent appendectomy for suspected appendicitis recurrence after initial antibiotic treatment did so within 1 year after randomization.

To our knowledge, there are no available long-term results of previous randomized trials comparing the overall costs of antibiotic therapy versus appendectomy in the treatment of uncomplicated acute appendicitis. This study reports the 5-year overall costs for all the patients enrolled in the original APPAC (APPendicitis ACuta) trial.

## Methods

### Study design

Details of the study design, rationale and methods have been published previously.[6, 18] The initial APPAC trial was a multicentre, open-label, randomized clinical non-inferiority trial conducted at six Finnish hospitals (Turku, Oulu, and Tampere University hospitals, and Jyväskylä, Mikkeli, and Seinäjoki Central hospitals). The trial protocol[18] was approved by the ethics committees of all participating hospitals. Trial was registered in clinicaltrials.gov: NCT01022567.

https://clinicaltrials.gov/ct2/show/NCT01022567

### Participants

Patients aged 18 to 60 years admitted to the emergency department with a clinical suspicion of uncomplicated acute appendicitis confirmed by CT were enrolled in the study after giving written informed consent. CT criteria for acute appendicitis included appendiceal diameter exceeding 6 mm with wall thickening accompanied with at least one of the following features: abnormal contrast enhancement of the appendiceal wall, inflammatory edema, or minor fluid collections around the appendix. Exclusion criteria included complicated acute appendicitis defined as the presence of an appendicolith, perforation, abscess, or suspicion of a tumor on the CT scan. Other exclusion criteria were contraindications for CT, peritonitis, unable to co-operate and provide informed consent, and the presence of serious systemic illness.

### Procedures

For patients randomized to operative treatment, the predefined surgical procedure in the trial protocol was open appendectomy performed using a McBurney right lower quadrant muscle-splitting incision technique. Laparoscopic appendectomy was performed in 15 patients (5.5 per cent). Prophylactic antibiotics (single dose of cefuroxime 1.5g and metronidazole 500mg intravenously) were administered approximately 30 minutes before incision.

For patients randomized to antibiotic therapy, intravenous ertapenem sodium (1g/day) was administered for 3 days followed by 7 days of oral levofloxacin (500mg once daily) and metronidazole (500mg 3 times daily).

### Outcomes

The primary endpoint was treatment success predefined to be assessed at one-year follow-up.[6, 18] In the antibiotic group it was defined as resolution of acute appendicitis resulting in discharge from the hospital without the need for surgical intervention and no recurrent appendicitis during a minimum follow-up of one year. In the appendectomy group treatment efficacy was defined as a patient successfully undergoing an appendectomy.

The predefined secondary endpoints included late recurrence (after 1 years) of acute appendicitis after antibiotic treatment, overall postintervention complications, length of hospital stay, the amount of sick leave, postintervention pain scores (VAS, visual analogue scale), and treatment costs.[18] According to the study protocol, all patients with clinically suspected recurrent appendicitis during follow-up underwent appendectomy without further imaging. The present study focuses on all secondary outcomes affecting the overall costs in order to evaluate the economic consequences of the both randomized treatment options at 5-year follow-up.

### Cost analysis

All cost estimates were based on the cost levels of 2016. In the base case of analyses annual discount rate of 5 per cent was applied to all costs. Hospital charges were recorded based on diagnosis-related group codes as overall hospital costs and registered in all participating hospitals. The hospital charges were a bulk sum including all the cost components (operation related costs, specialist fees, medicine, accommodation, food etc.) incurred by the treatment and patient up-keep during hospitalization, thus representing the true costs used to charge the final payer. In the Finnish system, the community of residence of the patient pays the diagnosis-related group-based bulk costs charged by the hospitals and accurate proportion of each separate component cannot be reliably identified. In Finland the health care system is organised by communities, based on central government guidance and hospital costs are charged from communities in full.

The treatment of acute appendicitis in Finland is practically entirely carried out in the public hospital setting, and the role of occasional rare involvement of private health care providers or primary health care organizations is marginal and has insignificant economic impact. In our analysis, all major hospital costs were recorded, whether generated by the initial visit and subsequent treatment or possible complications or recurrent appendicitis during the 5-year follow-up period, and an intention-to-treat analysis was performed.

Two antibiotic group patients undergoing subsequent surgery during the long-term follow-up were lacking sufficient data regarding operative treatment or productivity loss information based on surgical treatment abroad in one patient and change in hospital cost recording system in the other patient, but they were also included in the analyses. Their follow-up costs were estimated by using age and sex standardized linear regression models, based on complete initial operation and productivity loss data from the hospital district where they received their initial treatment.

When estimating the costs of absence from work, the human capital approach was applied. The days spent in hospital were all considered sick leave days and additional sick leave prescribed at discharge was also recorded. The costs of productivity losses were based on the average monthly gross salaries for working Finnish adults in 2016, €3075 for women and €3675 for men. The per day productivity loss estimate was computed by dividing the gross monthly salary by 21, the number of average monthly working days.

The cost for imaging, laboratory, and medicine used during hospitalization or prescribed at hospital discharge were marginal and non-significant at 1-year follow-up.[16] Therefore this data was not collected for the long-term follow-up at five years as omitting these cost components was not expected to have any influence on the comparison outcome between the two treatment alternatives.

### Statistical analysis

Categorical variables were described using frequencies and percentages, and continuous variables with means and 95% confidence intervals (95% CI) or in case of skewed variables medians with 95% CI. Statistical analysis of the data on average costs was based on Student’s *t-* test. The data on hospital charges, productivity costs and overall costs had very acceptable skewness (.77,.71 and .61) and kurtosis (.93, 1.51 and 1.02, respectively) values and the Student’s t-test was concluded robust enough to minor violation of the normality assumption. Differences between groups in length of hospital stay and sick leave were tested using Mann-Whitney U–test because of very skewed distributions. Normality of the distributions was evaluated using Kolmogorov-Smirnov–test, skewness and kurtosis of the distributions and visual evaluation.

Sensitivity analyses were performed to determine whether the final outcome was sensitive to certain crucial factors. The values of selected components were changed and the effect on the outcome estimate was evaluated. The role of the costs of absence from work days was determined in two directions, i.e. by decreasing the days of prescribed sick leave and increasing the salary costs with 10% intervals up to 50% lower and higher values. When estimating the sensitivity of sick leave days, the days in hospital were not reduced, only the sick leave days prescribed when the patient was discharged. The effect of discount rate was evaluated by performing the analyses using also 0%, 3%, 7% and 10% annual rates. Two-sided p-values less than 0.05 were considered statistically significant. All analyses were performed using SPSS software version 23 (IBM, Armonk, New York, USA).

## Results

**Fig 1:**
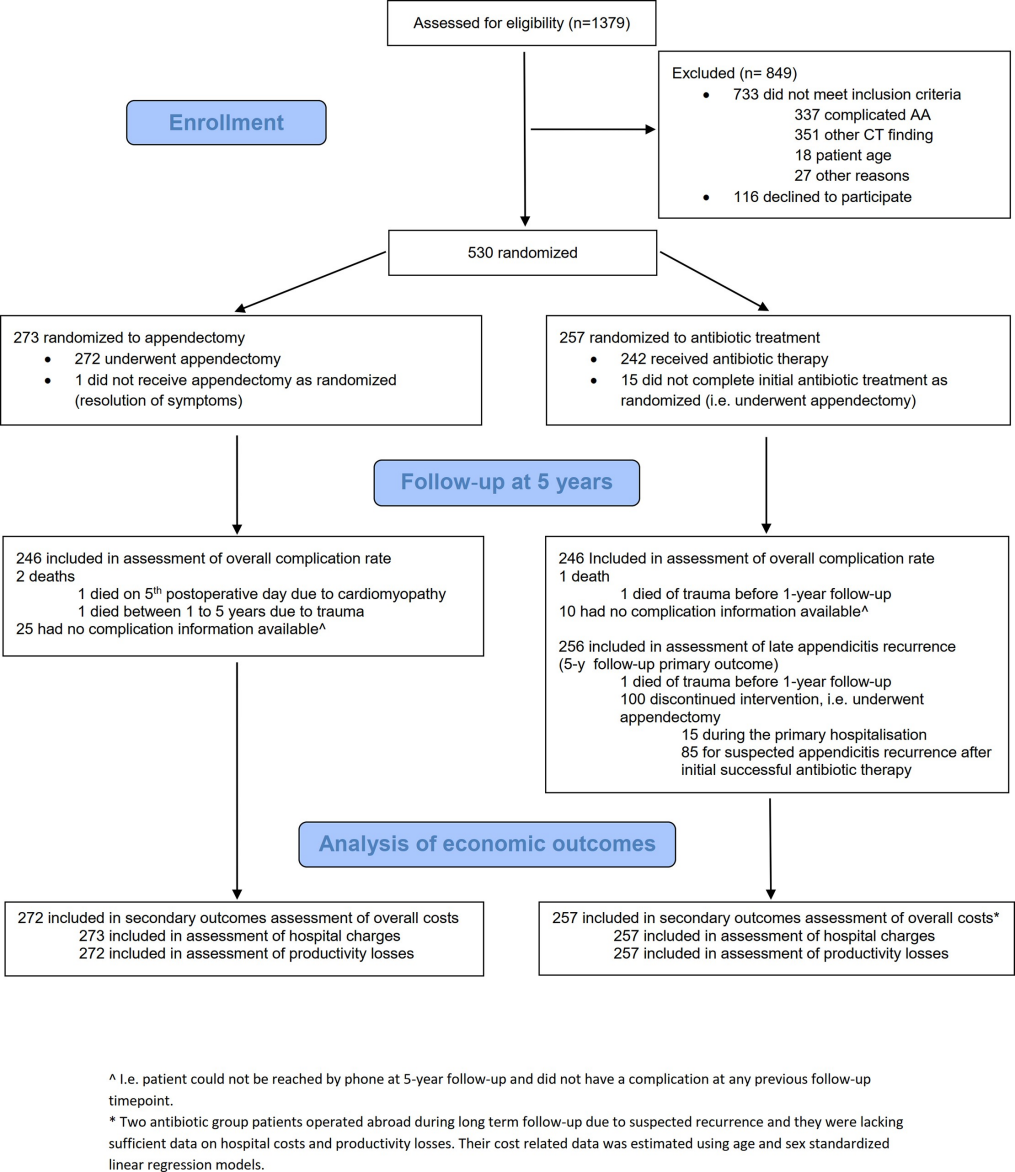
Path of patients in the APPendicitis ACuta (APPAC) trial.

Between November 10, 2009, and June 20, 2012, 530 patients were enrolled, and 273 patients were randomized to the appendectomy group and 257 patients to antibiotic treatment. Fig 1 shows the trial profile; 529 patients out of 530 were included in the economic analysis at 5 years excluding only the patient in the antibiotic group, who died of trauma before 1-year follow-up time point. Out of 530 patients, 495 (93%) were reached by telephone for follow-up at five years and hospital records were checked for all patients. Results for the main long-term follow-up endpoint of late appendicitis recurrence showed that at 5-year follow-up 61% (n = 156) of the antibiotic treatment group patients did not undergo appendectomy.[13] At five years, appendectomy group incurred significantly (p<0.001) higher overall costs (€5716; 95% CI 5510 to 5925) than antibiotic treatment group (€4171; 95% CI 3879 to 4463). The operative group overall costs were 1.4 times higher at five years with cost advantage of €1545 per patient (95% CI 1193 to 1899, p<0.001) for antibiotic therapy. In both groups the median length of hospital stay was 3 days (95% CI, 3 to 3). Patients in the operative group were prescribed more sick leave than those in the antibiotic group (median 22 (95% CI 19 to 23) versus 11 (11 to 12) days, respectively; p<0.001).

Higher costs in the surgical group were observed both in hospital charges and productivity losses. In both groups productivity losses formed the slightly larger proportion of the overall costs compared with hospital charges. Almost equal relative differences in the costs between the two study groups could be observed in hospital charges, productivity losses as well as in overall costs. The cost distribution is presented in detail in Table 1.

**Table 1:** Mean hospital charges, productivity losses and overall costs in Euros per patient for appendectomy and antibiotic therapy group patients with uncomplicated acute appendicitis at five-year follow-up.

	Appendectomy Group € (95% CI, €)	Antibiotic therapy Group € (95% CI, €)	Difference € (95% CI, €)	p<
**One-year follow-up**				
Hospital charges	2718 (2636–2799)	1707 (1547–1865)	1010 (835–1186)	0.001
Productivity losses	2962 (2806–3118)	1845 (1712–1976)	1117 (911–1322)	0.001
Overall costs	5680 (5489–5872)	3552 (3334–3769)	2127 (1840–2417)	0.001
**Five-year follow-up**				
Hospital charges	2730 (2645–2817)	2056 (1861–2251)	674 (465–883)	0.001
Productivity losses	2986 (2822–3149)	2115 (1950–2280)	871 (639–1104)	0.001
Overall costs	5716 (5510–5925)	4171 (3879–4463)	1545 (1193–1899)	0.001

The findings of this study were not sensitive to any of the selected components studied. The sensitivity analysis of the costs for the two groups after 5-year follow-up is presented in Table 2. They remained in the range of 1.3 to 1.4 times higher costs for operative group, even when the most extreme value options were applied.

**Table 2:** Sensitivity analyses of mean overall costs of appendectomy and antibiotic therapy group patients with uncomplicated acute appendicitis in Euros per patient at five-year follow-up.

	Appendectomy Group € (95% CI, €)	Antibiotic Therapy Group € (95% CI, €)	Difference € (95% CI, €)	p<
**Discount rate**				
10%	5716 (5510–5922)	4137 (3853–4423)	1579 (1229–1926)	0.001
0%	5720 (5511–5928)	4212 (3911–4513)	1508 (1145–1870)	0.001
**Sick leave days**				
30% fewer	4951 (4784–5119)	3728 (3460–3995)	1223 (911–1534)	0.001
50% fewer	4438 (4296–4581)	3406 (3158–3653)	1032 (752–1313)	0.001
**Salary costs**				
30% higher	9335 (9030–9638)	6538 (6102–6972)	2797 (2271–3321)	0.001
50% higher	9932 (9599–10265)	6964 (6506–7422)	2968 (2407–3527)	0.001

## Discussion

The overall costs at 5-year follow-up were 1.4 times higher in the appendectomy group than in the antibiotic group when all major costs were taken into account, whether generated by the initial visit and subsequent treatment or possible recurrent appendicitis. After the first year of follow-up, overall costs in the appendectomy group were 1.6 times higher[16] and despite the additional 30 antibiotic group patients subsequently undergoing appendectomy due to suspected recurrent appendicitis between 1 and 5 years, the cost difference in favour of antibiotic treatment remained significant. These long-term results on costs combined with the finding that appendicitis recurrence rate seems to markedly diminish after the first year, suggest that treating uncomplicated acute appendicitis with antibiotics instead of appendectomy results in lower overall costs also at even longer-term follow-up exceeding 5 years.

These results corroborate the findings from the decision tree model by Wu et al.[17] and earlier reports in children as well.[19, 20] In a retrospective cohort study on adults with a mean follow-up of 3.2 years, Sceats et al.[21] reported contradictory results with slightly higher treatment costs for antibiotic treatment compared with operative approach for uncomplicated acute appendicitis. However, they only included the direct treatment costs in their analysis[21] creating a major bias in the overall cost assessment as sick leave has been estimated to be the single most expensive component of costs in general practice.[22] This is supported by the current study showing that in both treatment groups the costs of productivity losses estimated by valuing the sick leave days were higher than the hospital charges. Due to the Finnish liability legislation regarding sick leave, patients mainly use the entire prescribed sick leave making the length of sick leave as the most feasible and reliable variable to use for the productivity loss calculations.

Surgeons have very different views of the optimal number of sick leave days for the same patient cases.[23] From a societal perspective this variability can markedly affect the potential cost savings as productivity losses are a key cost component. In this study at 5-year follow-up the antibiotic group patients were prescribed significantly less sick leave compared to patients in the operative group. Especially at the time of the APPAC trial patient enrolment, the non-operative treatment of uncomplicated acute appendicitis was not a familiar or generally accepted treatment alternative to appendectomy among surgeons relating also to assessing the length of the sick leave for these patients. This is also relevant to the length of hospital stay; in the APPAC trial the hospital stay of minimum three days in the antibiotic group was predefined in the protocol to ensure patient safety, since neither the success nor safety of antibiotic treatment of uncomplicated acute appendicitis was known at the time of study initiation. This was the basis for the trial protocol decision to proceed to appendectomy in all antibiotic group patients with clinical suspicion of recurrent appendicitis. Recently, re-treatment of recurrent appendicitis with antibiotics has been described as a potential future treatment option, possibly even further decreasing the overall costs of non-operative treatment.[24]

As we now know that antibiotic therapy is safe[13, 25–27], both the sick leave and hospital stay duration could be substantially shortened in the future, presumably resulting in even further cost savings. In the NOTA study[28] the mean hospital stay was reported to be 0.4 days for patients treated with antibiotics and even a pilot study on outpatient management of uncomplicated acute appendicitis[29] has been successfully conducted in the US showing significantly lower costs incurred by patients treated with antibiotics compared to surgery. A significant future aim is to optimize the non-operative treatment of uncomplicated acute appendicitis. Optimizing the antibiotic therapy regarding the use of less broad-spectrum antibiotics, possibly avoiding i.v. administration and shorter duration of the antibiotic treatment [5] will also have a major influence on the length of hospital stay and consequently on the overall costs. In addition, a double-blinded, placebo-controlled RCT[7] will further assess symptomatic treatment alone compared with antibiotic therapy in the treatment of uncomplicated acute appendicitis.[14]

A limitation of the APPAC trial is the open approach in the surgery group as the current gold standard of laparoscopic appendectomy has been shown to shorten both the hospital stay and sick leave.[30, 31] Open appendectomy was chosen as the operative intervention based on both optimal standardization of the procedure and global generalizability given that laparoscopic equipment or expertise may not be available throughout the world.[18] However, the surgical costs related to laparoscopy are significantly higher compared to open approach and the open approach may not have a major impact on the treatment costs as similar total costs for patients treated with open and laparoscopic approaches were reported in a meta-analysis despite the shorter hospital stay associated with laparoscopy.[30] Moreover, Wu et al[17] reported initial antibiotic treatment incurring less costs compared to laparoscopic appendectomy in treating uncomplicated acute appendicitis. A strong element of this study is the high follow-up rate regarding all major costs as the hospital records of all patients were checked including the 35 patients lost-to follow-up interviews. The potential patient visits to their primary care doctors or private health care providers present a very marginal cost at maximum as in Finland it is extremely rare for patients to have significant surgical conditions or postoperative /postintervention symptoms treated somewhere else than at their local surgical hospital.

The Finnish cost estimates used in this study may differ from those observed in other societies with different salary levels or structures. However, even when the salary levels were increased by 50% in the sensitivity analysis, the differences between the groups remained similar. As the increase in the overall costs was almost equal in proportion to increases in the salary costs, these findings can be assumed to be generalizable also to societies with different salary levels or structures. People and societies tend to have a positive time preference—we prefer to have the benefits sooner and carry the costs later. Thus, costs that accrue in later years need to be valued differently than those that are formed earlier. This valuation is done by discounting, a method that produces net present values where costs accruing in different years are made comparable. Discount rate of 5% has become a standard in economic analysis over the years, although there is no scientific basis for using that particular rate. Thus, a sensitivity analysis is justified, where the discount rate is varied and the effect of changing it on the outcome measure is estimated. In the present study changing the discount rate had a non-significant and marginal effect mainly due to the low number of patients having recurrent appendicitis after the first year of follow-up.

In the APPAC trial, the treatment costs at 1-year follow-up were 1.6 times higher in the appendectomy group[16] and remained 1.4 times higher at 5-year follow-up as the majority of recurrences after antibiotic treatment occurred within the first year. In our earlier report at 5-year follow-up[13], we reported a cumulative incidence of recurrent appendicitis at 1, 2, 3, 4, and 5 years of 27.3% at 1 year, 34.0% at 2, 35.2% at 3, 37.1% at 4, and 39.1% at 5 years with only 13 out of 85 appendectomies for suspected recurrence performed during the years 3 to 5. This finding of the recurrence rate markedly declining after the second year most likely results in this study to represent quite an accurate evaluation of even longer-term cost analysis of antibiotic therapy for uncomplicated acute appendicitis. The optimal treatment paradigm of uncomplicated acute appendicitis is quite complex, demanding the clinicians to consider multiple factors including patient preference when evaluating the optimal treatment for each patient.[32] The earlier data on the feasibility and safety of antibiotic therapy in the treatment of CT-scan confirmed uncomplicated acute appendicitis[8–10, 13] can now be corroborated by these results suggesting that antibiotic treatment could be the treatment alternative incurring least costs also at long-term follow-up. Although economic factors must not be the decisive element when choosing the treatment for an individual patient, the costs of different treatment alternatives need to be considered. As uncomplicated acute appendicitis is globally one of the most common surgical conditions[1], the potential cost savings of even a partial treatment paradigm shift from appendectomy towards antibiotics alone could be significant.

## Supporting information

S1 File APPAC Study protocol and statistical analysis plan. APPAC study protocol and statistical analysis plan.
(DOCX)
