# Supplementary material for: Cost analysis of antibiotic therapy versus appendectomy for treatment of uncomplicated acute appendicitis: 5-year results of the APPAC randomized clinical trial
Source: PLoS One. 2019 Jul 25;14(7):e0220202. doi: 10.1371/journal.pone.0220202 (PMC6657874; doi:10.1371/journal.pone.0220202)
Supplement: S1 File — APPAC study protocol and statistical analysis plan. (DOCX) [file pone.0220202.s001.docx]

**APPAC trial: statistical analysis plan (original)**

Paulina Salminen, M.D., Ph.D., Hannu Paajanen, M.D., Ph.D., Tero Rautio, M.D., Ph.D., Saija Hurme, M.Sc., and Juha M. Grönroos, M.D., Ph.D.,

for the Finnish APPAC group

**1. Introduction**

The APPAC trial is a randomized prospective controlled, open label, non-inferiority multicenter trial designed to compare antibiotic therapy (ertapenem) with emergency appendectomy in the treatment of uncomplicated AA. The primary endpoint of the study is the success of the randomized treatment. In the antibiotic treatment arm successful treatment is defined as being discharged from the hospital without the need for surgical intervention and no recurrent appendicitis during a follow-up of one-year (treatment efficacy). Treatment efficacy in the operative treatment arm is defined as successful appendectomy evaluated to be 100 %. Secondary endpoints are post-intervention complications, overall morbidity and mortality, the length of hospital stay and sick leave, treatment costs and pain scores (VAS, visual analoque scale).

**2. Sample size calculation**

The sample size calculation of the trial was based on the self-evident fact that the efficacy of appendectomy as a treatment for AA is 100 %, but antibiotic therapy will not provide adequate source control in all patients with uncomplicated AA. However, the hypothesis of the APPAC trial is that operative treatment of uncomplicated AA is not mandatory for the majority of patients as 75 – 85 % of patients with uncomplicated AA can be cured with wide-spectrum antibiotics avoiding a large number of unnecessary appendectomies [1]. For the primary endpoint of treatment success for the randomized therapy tested in a randomized, controlled, open label, non-inferiority multicenter trial, we assumed 99 % healing rate of AA in the appendectomy group vs. 80 % success rate for the antibiotic therapy. A non-inferiority margin of 24 percentage points was used in the sample size calculations meaning that the lower limit of the success in antibiotic therapy would be 75 %. We calculated that a sample size of 275 patients per group would give a power of 0.9 (1-β) to establish whether antibiotic treatment was not inferior to appendectomy evaluated by treatment success in both study arms (significance level of 0.05 α). With an estimated 10 percent of the trial patients lost to follow-up, a maximum of 610 patients will be enrolled.

**3. Interim analyses**

To ensure the safety of the antibiotic treatment the frequency of the treatment efficacy will be examined after randomizing 150-200 patients. No statistical tests will be conducted at this point.

**4. Statistical analysis**

Categorical variables will be characterized by treatment using frequencies and percents and for continuous variables means and standard deviations or medians and range will be used. Two-tailed p-values will be used and p-values less than 0.05 will be considered statistically significant. The main analyses will be based on the intention-to-treat principle. Missing data will be excluded from the analyses. Statistical analyses will be performed using SAS System for Windows, Version 9.2 (SAS Institute Inc., Cary, NC).

**4.1. Primary outcome**

Primary endpoint of the study is treatment efficacy and groups will be compared using equivalence trial setting. For comparing treatments the 95% confidence intervals for treatment efficacy will be presented.

**4.2. Secondary outcomes**

For the secondary endpoints data will be compared as superiority trial setting and in superiority tests a two-tailed P value ≤ 0.05 will be considered statistically significant. For categorical variables Pearson’s Chi Squared test will be used to test difference between groups. Differences between groups for normally distributed variables will be tested using independent sample t-test and for non-normally distributed variables Mann-Whitney U- test will be used. Normality tests will be performed to justify the analyses.

**4.3. Subgroup analyses**

Additional analyses will be performed for overall morbidity using per-protocol principle.

**5. Cost analysis**

All related costs will be estimated based on the actual input terms of resource use and personnel in the 12-month follow-up period after randomization. All costs will be derived from the Finnish hospital cost or determined in co-operation with the hospital administration. Direct medical costs will be recorded in the case record forms. Indirect costs arising from losses in productivity will be assessed by means of the Health and Labor questionnaire and will be calculated by means of the friction cost method.

**6. Follow-up**

The main results of the trial will be reported after one year follow-up of the patients. After that the patients will be interviewed also at 3, 5 and 10 years after treatment and the data is evaluated in every time-point.

**References**

1. Mason, R.J., A. Moazzez, H. Sohn, and N. Katkhouda, Meta-analysis of randomized trials comparing antibiotic therapy with appendectomy for acute uncomplicated (no abscess or phlegmon) appendicitis*.* Surg Infect (Larchmt), 2012. 13(2): p. 74-84.

**APPAC trial: statistical analysis plan (final)**

Paulina Salminen, M.D., Ph.D., Hannu Paajanen, M.D., Ph.D., Tero Rautio, M.D., Ph.D., Pia Nordström, M.D., Ph.D., Markku Aarnio, M.D., Ph.D., Tuomo Rantanen, M.D., Ph.D., Risto Tuominen, M.P.H., Ph.D, Saija Hurme, M.Sc., Johanna Virtanen, M.D., Jukka-Pekka Mecklin, M.D., Ph.D., Juhani Sand, M.D., Ph.D., Airi Jartti, M.D., Irina Rinta-Kiikka, M.D., Ph.D., and Juha M. Grönroos, M.D., Ph.D.,

for the Finnish APPAC group

From the Division of Digestive Surgery and Urology, Departments of Acute and Digestive Surgery, Turku University Hospital and The Department of Surgery, Turku University, Turku (P.S., J.M.G.), the Department of Surgery, Mikkeli Central Hospital, Mikkeli (H.P.) and Institute of Clinical Medicine, University of Eastern Finland (H.P., J-P.M., T. Rantanen), the Department of Surgery, Oulu University Hospital, Oulu (T. Rautio), Division of Surgery, Gastroenterology and Oncology, Tampere University Hospital, Tampere (P.N., J.S.), Department of Surgery, Jyväskylä Central Hospital, Jyväskylä (M.A., J-P.M.), Department of Surgery, Kuopio University Hospital, Kuopio and the Department of Surgery, Seinäjoki Central Hospital, Seinäjoki (T. Rantanen), Department of Public Health, University of Turku, and Primary Health Care Unit, Hospital District of Southwest Finland (R.T.), Department of Biostatistics, University of Turku, Turku (S.H.), Department of Radiology, Turku University Hospital, Turku (J.V.), Department of Radiology, Oulu University Hospital, Oulu (A.J.), Department of Radiology, Tampere University Hospital, Tampere (I.R-K.) – all in Finland.

**1. Introduction**

The APPAC trial is a randomized prospective controlled, open label, non-inferiority multicenter trial designed to compare antibiotic therapy (ertapenem) with emergency appendectomy in the treatment of uncomplicated AA. The primary endpoint of the study is the success of the randomized treatment. In the antibiotic treatment arm successful treatment is defined as being discharged from the hospital without the need for surgical intervention and no recurrent appendicitis during a follow-up of one-year (treatment efficacy). Treatment efficacy in the operative treatment arm is defined as successful appendectomy evaluated to be 100 %. Secondary endpoints are post-intervention complications, overall morbidity and mortality, the length of hospital stay and sick leave, treatment costs and pain scores (VAS, visual analoque scale).

**2. Sample size calculation**

The sample size calculation of the trial was based on the self-evident fact that the efficacy of appendectomy as a treatment for AA is 100 %, but antibiotic therapy will not provide adequate source control in all patients with uncomplicated AA. However, the hypothesis of the APPAC trial is that operative treatment of uncomplicated AA is not mandatory for the majority of patients as 75 – 85 % of patients with uncomplicated AA can be cured with wide-spectrum antibiotics avoiding a large number of unnecessary appendectomies [1]. For the primary endpoint of treatment success for the randomized therapy tested in a randomized, controlled, open label, non-inferiority multicenter trial, we assumed 99 % healing rate of AA in the appendectomy group vs. 80 % success rate for the antibiotic therapy. A non-inferiority margin of 24 percentage points was used in the sample size calculations meaning that the lower limit of the success in antibiotic therapy would be 75 %. We calculated that a sample size of 275 patients per group would give a power of 0.9 (1-β) to establish whether antibiotic treatment was not inferior to appendectomy evaluated by treatment success in both study arms (significance level of 0.05 α). With an estimated 10 percent of the trial patients lost to follow-up, a maximum of 610 patients will be enrolled.

**3. Interim analyses**

To ensure the safety of the antibiotic treatment the frequency of the treatment efficacy will be examined after randomizing 150-200 patients. No statistical tests will be conducted at this point.

**4. Statistical analysis**

Categorical variables will be characterized by treatment using frequencies and percents and for continuous variables means and standard deviations or medians and range will be used. Two-tailed p-values will be used and p-values less than 0.05 will be considered statistically significant. The main analyses will be based on the intention-to-treat principle. Missing data will be excluded from the analyses. Statistical analyses will be performed using SAS System for Windows, Version 9.2 (SAS Institute Inc., Cary, NC).

**4.1. Primary outcome**

Primary endpoint of the study is treatment efficacy and groups will be compared using equivalence trial setting. For comparing treatments the 95% confidence intervals for treatment efficacy will be presented.

**4.2. Secondary outcomes**

For the secondary endpoints data will be compared as superiority trial setting and in superiority tests a two-tailed P value ≤ 0.05 will be considered statistically significant. For categorical variables Pearson’s Chi Squared test will be used to test difference between groups. Differences between groups for normally distributed variables will be tested using independent sample t-test and for non-normally distributed variables Mann-Whitney U- test will be used. Normality tests will be performed to justify the analyses.

**4.3. Subgroup analyses**

Additional analyses will be performed for overall morbidity using per-protocol principle.

In order to find predictive factors for a more complicated course of appendicitis a subgroup of patients with a complicated AA will be compared with the rest of the patients in antibiotic group. First the explorative analyses will be performed for pre-intervention variables (CRP, Hb, leukocyte, creatinine and pain) and if statistically significant differences between groups will be found, then ROC-analysis will be performed to find a cut-point value for variable in order to predict complicated AA. A ROC-curve, sensitivity and specificity will be presented as the results of analyses.

**5. Cost analysis**

All related costs will be estimated based on the actual input terms of resource use and personnel in the 12-month follow-up period after randomization. All costs will be derived from the Finnish hospital cost or determined in co-operation with the hospital administration. Direct medical costs will be recorded in the case record forms. Indirect costs arising from losses in productivity will be assessed by means of the Health and Labor questionnaire and will be calculated by means of the friction cost method.

**6. Follow-up**

The main results of the trial will be reported after one year follow-up of the patients. After that the patients will be interviewed also at 3, 5 and 10 years after treatment and the data is evaluated in every time-point.

**References**

1. Mason, R.J., A. Moazzez, H. Sohn, and N. Katkhouda, **Meta-analysis of randomized trials comparing antibiotic therapy with appendectomy for acute uncomplicated (no abscess or phlegmon) appendicitis***.* Surg Infect (Larchmt), 2012. **13**(2): p. 74-84.

**Summary of changes**

**Amendment for chapter 4.3. Subgroup analyses**

In order to find predictive factors for a more complicated course of appendicitis a subgroup of patients with a complicated AA will be compared with the rest of the patients in antibiotic group. First the explorative analyses will be performed for pre-intervention variables (CRP, Hb, leukocyte, creatinine and pain) and if statistically significant differences between groups will be found, then ROC-analysis will be performed to find a cut-point value for variable in order to predict complicated AA. A ROC-curve, sensitivity and specificity will be presented as the results.
